# 831. C-reactive Protein Trends in Burn Patients Following Skin Substitute Application

**DOI:** 10.1093/jbcr/irag033.270

**Published:** 2026-04-06

**Authors:** Gina L DeFelice, Hannah G Theriot, Denise M Danos, Jeffrey E Carter, Jonathan E Schoen, M Victoria P Miles

**Affiliations:** Burn Center at University Medical Center, New Orleans, LA; Louisiana State University Health Sciences Center, New Orleans, LA; Louisiana State University Health Sciences Center, New Orleans, New Orleans, LA; University Medical Center Burn Center, Louisiana State University Health Sciences Center New Orleans, New Orleans, LA; UCI Health Regional Burn Center, New Orleans, LA; University Medical Center Burn Center, Louisiana State University Health Sciences Center, Department of Surgery, New Orleans, New Orleans, LA

## Abstract

**Introduction:**

Burn injuries induce a profound hypermetabolic and inflammatory response that may persist long after the initial injury, impacting patient recovery. While early burn excision has been shown to mitigate this systemic inflammatory response, the role of skin substitute placement in attenuating patient metabolic demands remains unclear. This study evaluates if application of any skin substitute after burn excision impacts the C-reactive protein (CRP) trend in adult burn patients.

**Methods:**

A prospective observational analysis was performed at an ABA-verified burn center between April 29, 2025 and September 1, 2025. Adult patients were included if they sustained a thermal burn injury of any total body surface area (TBSA), underwent burn excision during hospitalization, and had one or more indirect calorimetry measurements using a metabolic cart performed during hospitalization. Data collected included patient demographics, burn mechanism, TBSA, anatomic location, timing and size of excision, type of skin substitute used, and serial CRP values. CRP trends in patients undergoing placement of skin substitute post-excision were modeled using mixed effects generalized linear regression assuming a lognormal distribution. Models were adjusted for age, sex, TBSA, inhalation injury, and concomitant trauma. Results are reported as relative rates (RR) and 95% confidence intervals (CIs).

**Results:**

A total of 19 patients met inclusion. The first skin substitute application occurred between hospital days 2 and 7, with a median of 4 days. Twelve patients received allograft, 5 received biodegradable temporizing matrix (BTM), 1 received monolayer matrix (MTX), and 1 received more than one skin substitute. CRP was compared over 4 weeks, and, at week 4, there was a 65% reduction in CRP compared to week 1 [RR (95% CI) = 0.35 (0.18,0.66); *p*=.0016].

**Conclusions:**

Skin substitute placement after burn excision was associated with a significant reduction in systemic inflammation, evidenced by a 65% decrease in CRP at week 4 compared to week 1. Although limited by a small sample size and heterogeneity of the substitute type, our results indicate that early wound coverage with skin substitute may help mitigate the hypermetabolic and inflammatory burden in burn injury. Timing and type of skin substitute remain an area of debate. Further studies are needed to clarify the timing, type, and metabolic impact of skin substitute application. Prospective data collection is ongoing.

**Applicability of Research to Practice:**

Despite the limited cohort, this study provides preliminary evidence supporting the potential metabolic benefits of skin substitute use after excision. These findings may guide future research on optimal timing and patient selection to improve outcomes in burn care.

**Funding for the study:**

Spirit of Charity Foundation Burn Fund.

